# Supplementary material for: Genetic deletion of mast cell serotonin synthesis prevents the development of obesity and insulin resistance
Source: Nat Commun. 2020 Jan 23;11:463. doi: 10.1038/s41467-019-14080-7 (PMC6978527; doi:10.1038/s41467-019-14080-7)
Supplement: Supplementary file 1 — Supplementary Information [file 41467_2019_14080_MOESM1_ESM.pdf]

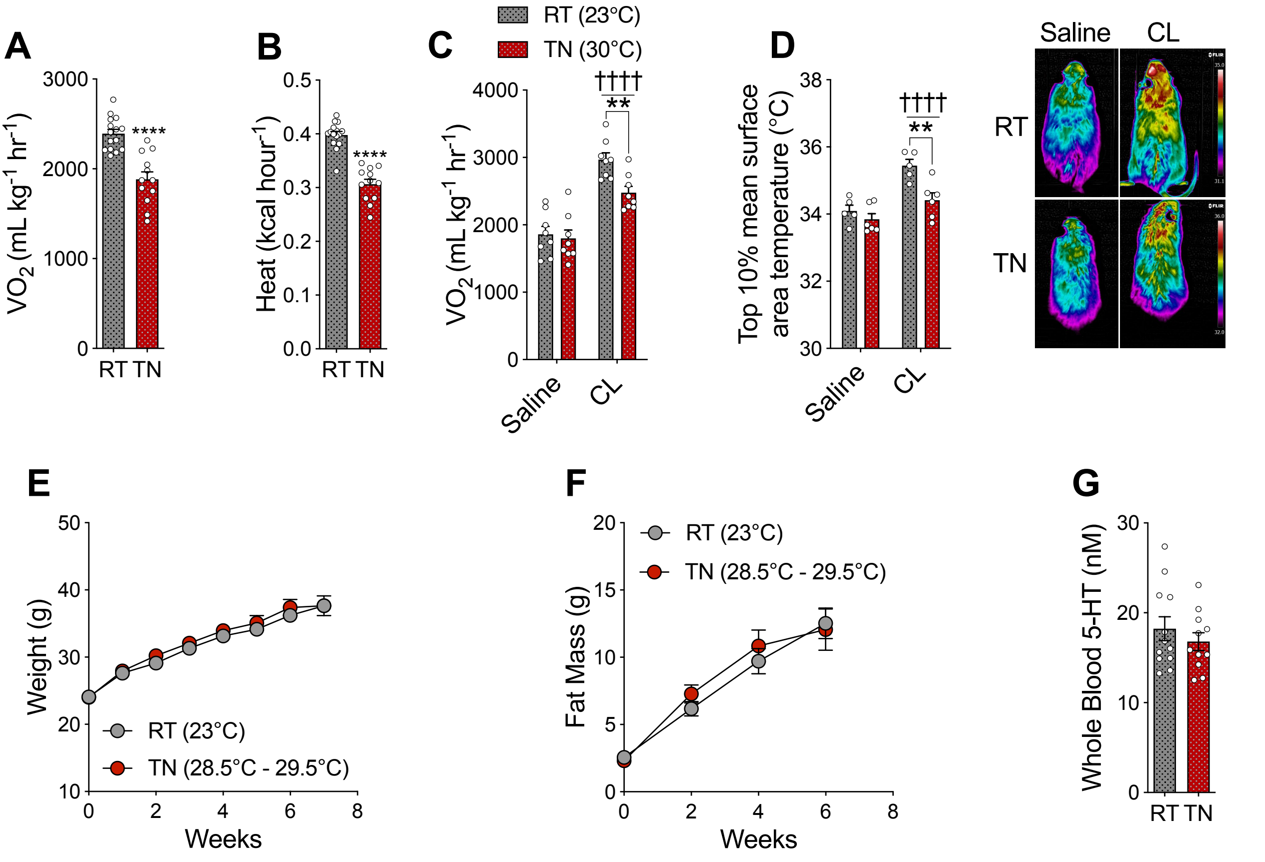

Supplementary Figure 1, Related to Figure 1: Thermoneutrality reduces BAT thermogenesis and whole body energy metabolism independent of changes in weight and circulating serotonin. (**A**) Oxygen consumption over 24h period and (**B**) Heat production over 24h period (n = 15 RT, 12 TN). (**C**) CL-induced oxygen consumption (n = 8). (**D**) CL-induced intrascapular temperature (n = 5 RT, 6 TN) with $^{††††}p$=0.0001 CL effect. (**E**) Weight gain (n = 23 RT, 11 TN) and (**F**) Fat mass (n = 12) in RT and TN housed mice over 7 weeks. (**G**) Whole blood serotonin (n = 12 RT, 11 TN) from sacrifice. Statistically significant effects ($^{**}p < 0.01$, $^{****}p < 0.0001$) determined by Student's t test, two-way ANOVA with Bonferroni post-test where appropriate. All data are expressed as mean ± SEM.

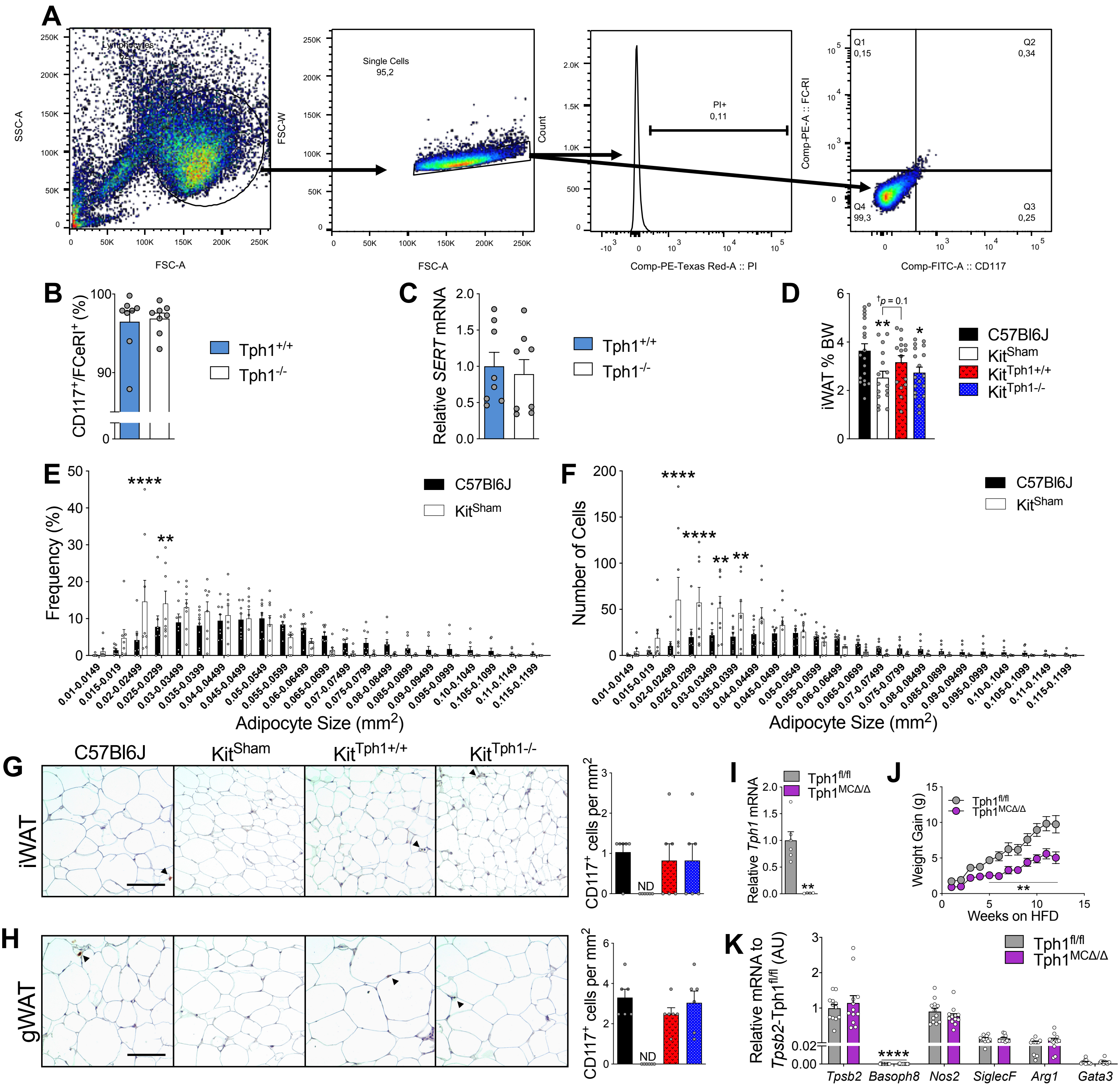

Supplementary Figure 2, Related to Figure 2 & 3: Characterization of in vitro-cultured mast cells and phenotyping of mice lacking mast cell serotonin synthesis. Gating strategy (**A**; detailed in Methods) of FACS analysis for mast cell purity ($CD117^+/Fc\varepsilon R1^+$) in $Tph1^{+/+}$ and $Tph1^{-/-}$ MCs (**B**) after 8 weeks of culture (n = 8). (**C**) Serotonin transporter (*SERT*) gene expression in $Tph1^{+/+}$ and $Tph1^{-/-}$ mast cells (n = 8). (**D**) % iWAT of body weight of C57BL6J (n = 19), Kit^{Sham} (n = 16), $Kit^{Tph+/+}$ (n = 15) and $Kit^{Tph-/-}$ (n = 18). (**E**) Frequency and (**F**) number of adipocyte sizes of C57BL6J and Kit^{sham} mice iWAT (n = 8). Representative (**G**) iWAT and (**H**) gWAT IHC images of mast cells using a $CD117^+$ antibody with respective quantification (n = 6). (**I**) *Tph1* expression in intraperitoneal lavage fluid (n = 6 $Tph1^{fl/fl}$, 4 $Tph1^{MC\Delta/\Delta}$). (**J**) Weight gain over 12 weeks (n = 12). (**K**) Gene expression of different immune cell markers implicated in influencing iWAT of $Tph1^{fl/fl}$ and $Tph1^{MC\Delta/\Delta}$ mice (n = 12). Statistically significant effects (*$p < 0.05$, **$p < 0.01$, ****$p < 0.0001$) determined by Student's t test, two-way ANOVA or two-way RM ANOVA with Bonferroni post-test where appropriate. All data are expressed as mean ± SEM.

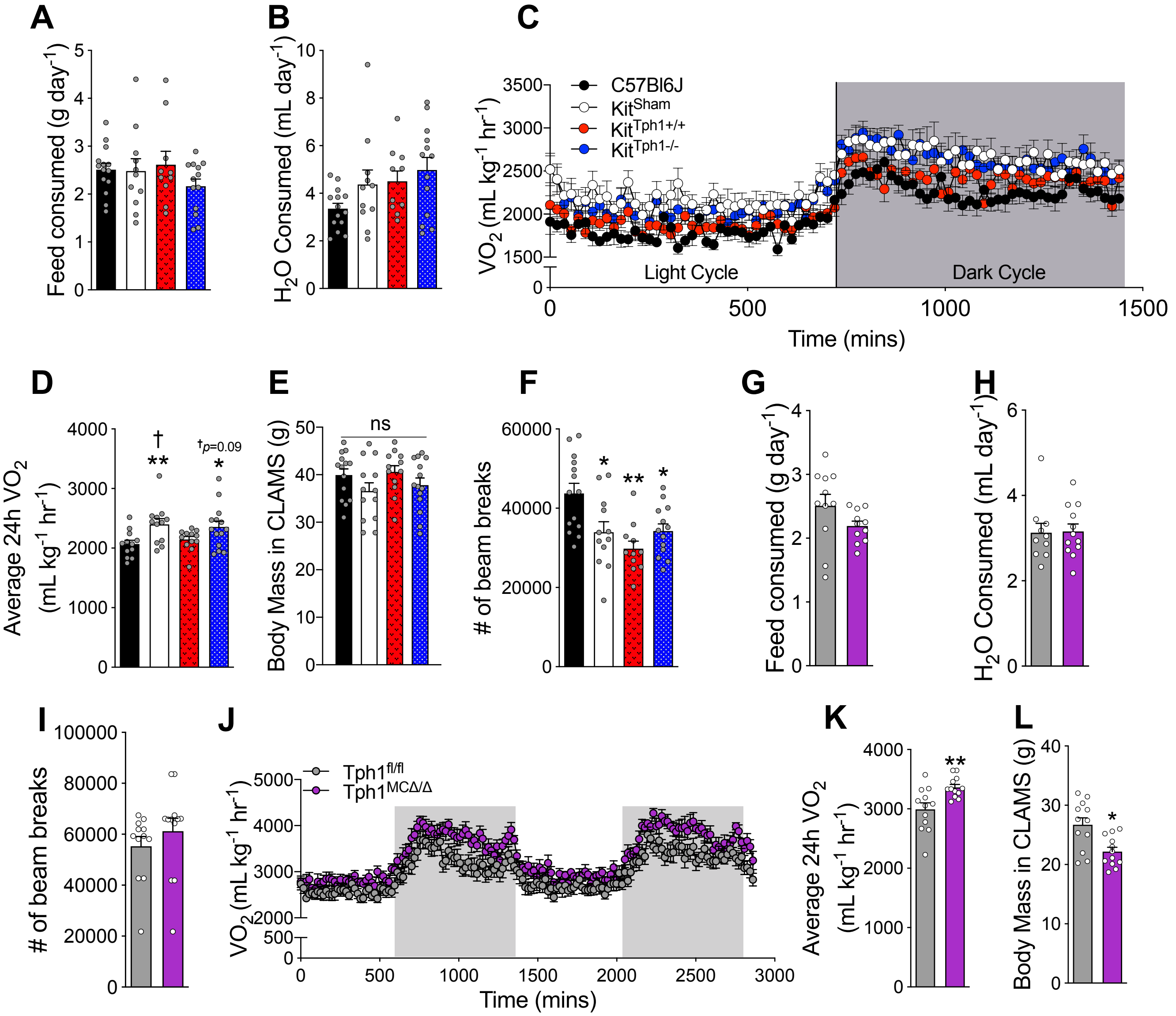

Supplementary Figure 3, Related to Figure 4: Energy balance of mouse models lacking mast cell serotonin. (**A**) Feed consumed in grams per day, (**B**) Water consumed per day, (**C**) Oxygen consumption over 24-hour period, (**D**) Average oxygen consumption over 24-hour period (**E**) Body mass of mice in CLAMS and (**F**) Activity levels as measured by beam breaks in metabolic cages over a 24-hour period of C57BL6J (n = 14), Kit^{Sham} (n = 11), $Kit^{Tph+/+}$ (n = 10) and $Kit^{Tph-/-}$ (n = 13). Statistically significant effects (*$p < 0.05$, **$p < 0.01$) of C57BL6J (*) and $Kit^{Tph1+/+}$ (†) between Kit^{Sham} and $Kit^{Tph1-/-}$ determined by one-way ANOVA with uncorrected Fisher's LSD post-test. (**G**) Feed consumed in grams per day (n = 11), (**H**) water consumed per day (n = 10 $Tph1^{fl/fl}$, 12 $Tph1^{MC\Delta/\Delta}$), (**I**) activity levels as measured by beam breaks in metabolic cages over a 24-hour period, (**J**) oxygen consumption over 48-hour period, (**K**) average oxygen consumption over 24-hour period and (**L**) body mass in clams of $Tph1^{fl/fl}$ and $Tph1^{MC\Delta/\Delta}$ mice (n = 12). Statistically significant effects (*$p < 0.05$, **$p < 0.01$) determined by Student's t test. All data are expressed as mean ± SEM.

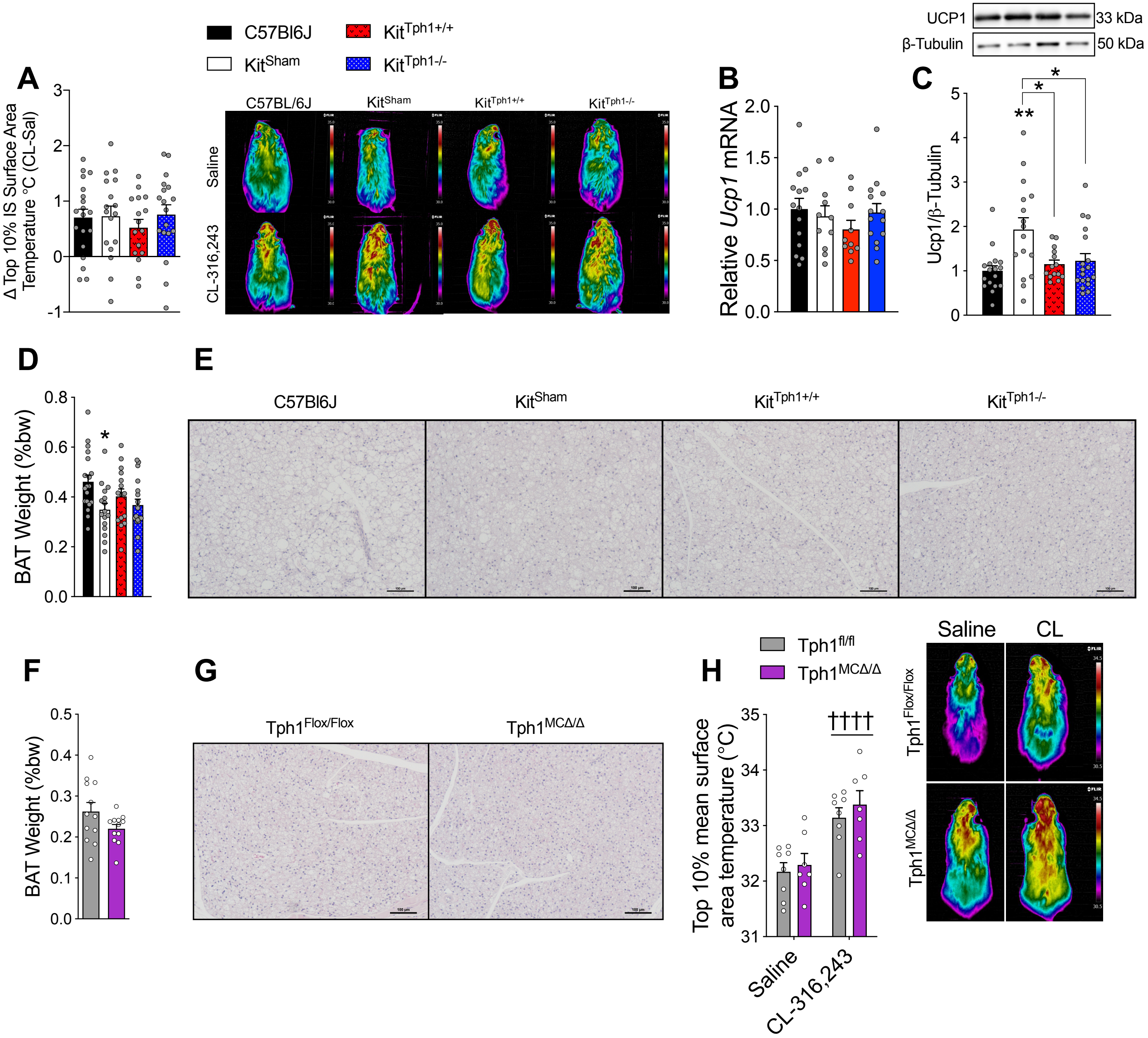

Supplementary Figure 4, Related Figure 4: Mice lacking mast cell serotonin exhibit little to no changes in BAT thermogenesis. (**A**) Delta top 10% intrascapular (IS) temperature between CL-316,243 and saline treatments with representative images in C57BL6J (n = 19), Kit^{Sham} (n = 17), $\text{Kit}^{\text{Tph+/+}}$ (n = 16) and $\text{Kit}^{\text{Tph-/-}}$ (n = 18). (**B**) *Ucp1* expression in C57BL6J (n = 14), Kit^{Sham} (n = 11), $\text{Kit}^{\text{Tph+/+}}$ (n = 10) and $\text{Kit}^{\text{Tph-/-}}$ (n = 14). (**C**) Ucp1 protein from in C57BL6J (n = 17), Kit^{Sham} (n = 18), $\text{Kit}^{\text{Tph+/+}}$ (n = 16) and $\text{Kit}^{\text{Tph-/-}}$ (n = 18). (**D**) BAT tissue weight in milligrams per gram of body weight and (**E**) Representative BAT H & E images with scale bars set at 100μm of C57BL6J (n = 19), Kit^{Sham} (n = 16), $\text{Kit}^{\text{Tph+/+}}$ (n = 15) and $\text{Kit}^{\text{Tph-/-}}$ (n = 18). Statistically significant effects ($^*p < 0.05$, $^{**}p < 0.01$) determined by one way ANOVA with uncorrected Fisher's LSD post-test. (**F**) BAT tissue weight in % body weight, (**G**) Representative BAT H & E, scale bars set to 100μm (n = 12), (**H**) Top 10% mean intrascapular surface area temperature with saline and CL-316,243 treatments (n = 8 $\text{Tph1}^{\text{fl/fl}}$, 7 $\text{Tph1}^{\text{MC}\Delta/\Delta}$) with representative thermal images of $\text{Tph1}^{\text{fl/fl}}$ and $\text{Tph1}^{\text{MC}\Delta/\Delta}$ mice. Statistically significant effects ($^{\dagger\dagger\dagger\dagger}p < 0.0001$) determined by two-way ANOVA with Bonferroni post-test. All data are expressed as mean ± SEM.

SUPPLEMENTARY TABLE

Table 1: Correlations between Tph1 and mast cell-related genes (bolded) generated from BioGPS gene annotation database.

Correlation	ID	Gene Symbol	Reporter
1	21990	Tph1	1419524_at
**0.9827**	**14125**	**Fcer1a**	**1421775_at**
**0.9812**	**26945**	**Tpsg1**	**1449564_at**
0.9683	16189	Il4	1449864_at
0.9664	19270	Ptprg	1434360_s_at
0.9649	17423	Ndst2	1417931_at
0.9602	74603	Cd200r3	1453813_at
**0.9601**	**12873**	**Cpa3**	**1448730_at**
**0.9595**	**17228**	**Cma1**	**1449456_a_at**
0.9543	17082	Il1rl1	1425145_at
0.9543	14126	Ms4a2	1421475_at
**0.9537**	**11689**	**Alox5**	**1441962_at**
**0.9513**	**235854**	**Mrgpra4**	**1451926_at**
**0.9512**	**16590**	**Kit**	**1452511_a_at**
0.9469	225192	Hrh4	1426099_at
0.9402	14723	Gp1ba	1422316_at
0.9399	67874	Rprm	142252_at
0.9345	214084	Slc18a2	1437079_at
0.9316	27384	Akr1c13	14119672_at
0.9252	73910	Arhgap18	146952_at
