# Supplementary material for: Genetic deletion of mast cell serotonin synthesis prevents the development of obesity and insulin resistance
Source: Nat Commun. 2020 Jan 23;11:463. doi: 10.1038/s41467-019-14080-7 (PMC6978527; doi:10.1038/s41467-019-14080-7)
Supplement: Supplementary file 3 — Reporting Summary [file 41467_2019_14080_MOESM3_ESM.pdf]

nature research

Corresponding author(s): Gregory Steinberg

Last updated by author(s): 12/11/2019

Reporting Summary

Nature Research wishes to improve the reproducibility of the work that we publish. This form provides structure for consistency and transparency in reporting. For further information on Nature Research policies, see Authors & Referees and the Editorial Policy Checklist .

Statistics

For all statistical analyses, confirm that the following items are present in the figure legend, table legend, main text, or Methods section.

n/a	Confirmed	
☐	☒	The exact sample size (*n*) for each experimental group/condition, given as a discrete number and unit of measurement
☐	☒	A statement on whether measurements were taken from distinct samples or whether the same sample was measured repeatedly
☐	☒	The statistical test(s) used AND whether they are one- or two-sided<br>*Only common tests should be described solely by name; describe more complex techniques in the Methods section.*
☒	☐	A description of all covariates tested
☐	☒	A description of any assumptions or corrections, such as tests of normality and adjustment for multiple comparisons
☐	☒	A full description of the statistical parameters including central tendency (e.g. means) or other basic estimates (e.g. regression coefficient) AND variation (e.g. standard deviation) or associated estimates of uncertainty (e.g. confidence intervals)
☒	☐	For null hypothesis testing, the test statistic (e.g. F, t, r) with confidence intervals, effect sizes, degrees of freedom and P value noted<br>*Give P values as exact values whenever suitable.*
☒	☐	For Bayesian analysis, information on the choice of priors and Markov chain Monte Carlo settings
☒	☐	For hierarchical and complex designs, identification of the appropriate level for tests and full reporting of outcomes
☒	☐	Estimates of effect sizes (e.g. Cohen's d, Pearson's r), indicating how they were calculated

Our web collection on statistics for biologists contains articles on many of the points above.

Software and code

Policy information about availability of computer code

Data collection	No software code was used in our study.
Data analysis	GraphPad Prism Version 8.1.0 (221) for data analysis and statistics, Microsoft Excel for Mac Version 16.27 for layout and analysis

For manuscripts utilizing custom algorithms or software that are central to the research but not yet described in published literature, software must be made available to editors/reviewers. We strongly encourage code deposition in a community repository (e.g. GitHub). See the Nature Research guidelines for submitting code & software for further information.

Data

Policy information about availability of data

All manuscripts must include a data availability statement. This statement should provide the following information, where applicable:

- Accession codes, unique identifiers, or web links for publicly available datasets
- A list of figures that have associated raw data
- A description of any restrictions on data availability

In our study, we used the BioGPS gene annotation portal (http://biogps.org/#goto=welcome) for the Tph1 gene with a correlation cutoff of 0.95 used in Figure 1 of our manuscript. There are no restrictions, to our knowledge, on this data's availability.

Field-specific reporting

Please select the one below that is the best fit for your research. If you are not sure, read the appropriate sections before making your selection.

☒ Life sciences ☐ Behavioural & social sciences ☐ Ecological, evolutionary & environmental sciences

For a reference copy of the document with all sections, see nature.com/documents/nr-reporting-summary-flat.pdf

Life sciences study design

All studies must disclose on these points even when the disclosure is negative.

Sample size	A sample size of 14 was determined by power calculation done from an initial pilot study done in C57BL6J mice and Ckitw-sh/w-sh mice on the same background. The total weight gained after 14 weeks of HFD was the primary outcome, which was significantly different between BL6 mice (18.98g) and CKitw-sh/w-sh (13.00g). With an alpha = 0.05, a desired power of 0.8 and a SD of ~5.5 (determined from average SD of weights of BL6 and Ckit groups), n = 14 was determined to be sufficient.<br><br>Sample sizes of greater than 9 were determined to be sufficient to observe at least a 20% reduction in our primary outcome (weight) in Tph1 knockout mice compared to WT, which was a significant reduction in our previous paper Crane et al. 2015 Nat Med between two groups (WT n=9, KO n=19). Therefore, experiments in Tph1 Mast cell knockout animals were done with a sample size of 9 or greater and extra mice were added to adjust for attrition.
Data exclusions	Data were excluded if the value was 2SD below or above the mean of the data set with ALL data points included.
Replication	Multiple cohorts were done with the same diet and in vivo testing protocol for all animal studies.
Randomization	Mice were randomly assigned to groups based on having equal body weight at the beginning of the study.
Blinding	Blinding was done for all in vivo tests. Experimenters did not know genotypes of mice during in vivo tests.

Reporting for specific materials, systems and methods

We require information from authors about some types of materials, experimental systems and methods used in many studies. Here, indicate whether each material, system or method listed is relevant to your study. If you are not sure if a list item applies to your research, read the appropriate section before selecting a response.

Materials & experimental systems

n/a	Involved in the study	
☐	☒	Antibodies
☒	☐	Eukaryotic cell lines
☒	☐	Palaeontology
☐	☒	Animals and other organisms
☒	☐	Human research participants
☒	☐	Clinical data

Methods

n/a	Involved in the study	
☒	☐	ChIP-seq
☐	☒	Flow cytometry
☒	☐	MRI-based neuroimaging

Antibodies

Antibodies used	Antibodies used were Anti-Mouse UCP1 (Alpha Diagnostics, 173435A4) and Anti-Mouse β-tubulin (Invitrogen, 322600), FCε-RI-PE (566608) and CD117-FITC (561680) antibodies (Becton Dickinson Canada Inc)
Validation	Mottillo et al., 2016 Cell Metabolism

Animals and other organisms

Policy information about studies involving animals; ARRIVE guidelines recommended for reporting animal research

Laboratory animals	Mice from Jackson Laboratories: Male-C57BL6J (000664), Male-CKitW-sh/W-sh (012861), Male/female-Cpa3-Cre (026828). From Gerard Karsenty: Male/female Tph1tm1Kry (MGI: 3837399). Tph1KO and WT Littermates: (Crane et al., 2015 Nat Med) All on C57BL6 Background
Wild animals	Study did not involve wild animals
Field-collected samples	Study did not involve samples collected from the field.
Ethics oversight	All animal experiments were performed in accordance with the McMaster Animal Care Committee guidelines and conducted under the Canadian guidelines for animal research (AUP: 16-12-41).

Note that full information on the approval of the study protocol must also be provided in the manuscript.

Flow Cytometry

Plots

Confirm that:

- [x] The axis labels state the marker and fluorochrome used (e.g. CD4-FITC).
- [x] The axis scales are clearly visible. Include numbers along axes only for bottom left plot of group (a 'group' is an analysis of identical markers).
- [x] All plots are contour plots with outliers or pseudocolor plots.
- [x] A numerical value for number of cells or percentage (with statistics) is provided.

Methodology

Sample preparation	Bone marrow-derived Mast cells were cultured in vitro
Instrument	FACSCanto (BD Biosciences)
Software	FlowJo software 10.6.0
Cell population abundance	On average, the Mast cell population was greater than 95% as shown by Supplementary Figure 2A in the manuscript
Gating strategy	Manual gating of high density areas ranging from 110k-250k FSC and 30k-180k SSC were initially performed on in vitro cultured mast cells. This was followed by another manual gating strategy to identify CD117+ and FCeRI+ positive cells. Gating strategy is explained in more detail in the Methods Section.

- [x] Tick this box to confirm that a figure exemplifying the gating strategy is provided in the Supplementary Information.
